# Pulmonary megakaryocytes orchestrate host defense via PcrV-induced NF-κB signaling in *Pseudomonas aeruginosa *infection

**DOI:** 10.1186/s12964-026-03072-y

**Published:** 2026-07-20

**Authors:** Xiaolong Guo, Qian Dai, Xiping Liao, Yannan Ma, Hengqian Liu, Junzhi Xiong, Hua Yu, Yuanyuan Li, Kebin Zhang, Jianyun Zhou, Hongli Zhou

**Affiliations:** 1 Clinical Medical Research Center, the Second Affiliated Hospital, Army Medical University, Chongqing, 400037 China; 2 Laboratory Medicine Department, Zigong Fourth People’s Hospital, Sichuan, 643000 China; 3 Department of Gastroenterology, the Second Affiliated Hospital, Army Medical University, Chongqing, 400037 China; 4 School of Medicine, Chongqing University Cancer Hospital, Chongqing University, Chongqing, 400030 China

**Keywords:** Megakaryocytes, Bacterial pneumonia, Immune modulator, *Pseudomonas aeruginosa*, PcrV

## Abstract

**Background:**

Megakaryocytes, beyond their classical role in platelet formation, are increasingly recognized as immune modulators. This study aimed to investigate the function and activation mechanisms of megakaryocytes during *Pseudomonas aeruginosa* (PA) infection, focusing on their interaction with bacterial toxins and innate immune pathways.

**Methods:**

This study evaluated the response of pulmonary megakaryocytes to PA-induced acute pneumonia in mice. Specifically, the activation status of pulmonary megakaryocytes was determined by flow cytometry and sorting, followed by RNA sequencing, combining Gene Ontology and Gene Set Enrichment Analysis. The expression of pro-inflammatory cytokines and chemokines within megakaryocytes after PA infection were detected by qPCR and fluorescent confocal imaging. The effect of megakaryocytes on the chemotaxis and recruitment of neutrophils and monocytes was demonstrated using chemotaxis assays. Additionally, purified PA toxins were injected into the lungs of mice in vivo or added to flow-sorted cells in vitro in order to induce inflammation and evaluate the response of megakaryocytes to a single PA toxin. The activation status of intracellular signaling pathways was verified through inhibitor-mediated blocking experiments.

**Results:**

PA infection significantly activates megakaryocytes, which promote the recruitment of phagocytes and enhance the host’s defense against PA infection. The innate immune response in the lungs was diminished during the early stages of PA infection among the megakaryocytes depleted mice, resulting in increased bacterial load and higher mortality. Flow cytometry sorting followed by RNA sequencing indicated that megakaryocytes exhibit a typical pro-inflammatory phenotype, along with NF-κB pathways activation. Furthermore, the PA toxin PcrV was identified as a key NF-κB activator on megakaryocytes, as demonstrated by increased expression of inflammatory markers and chemokines in response to PcrV. Inhibition of NF-κB signaling attenuated this pro-inflammatory phenotype of megakaryocytes.

**Conclusions:**

This study provides new insights into how megakaryocytes shape the cytokine and chemokine landscape in the lung during PA infection. It highlights the intrinsic interactions between megakaryocytes and phagocytes during pathogen invasion, suggests that managing megakaryocyte activity could be a new strategy to enhance immune defense.

**Graphical Abstract:**

**Figure:**
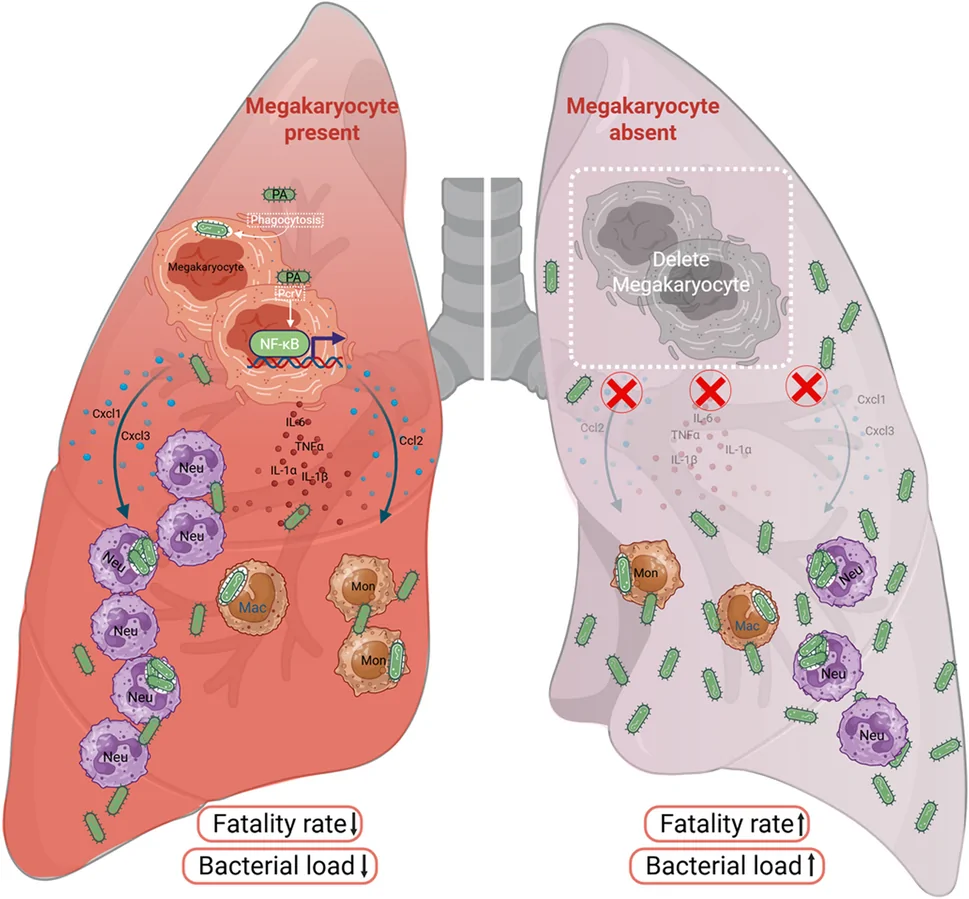

**Supplementary Information:**

The online version contains supplementary material available at https://doi.org/10.1186/s12964-026-03072-y.

## Abstract (plain language summary)

Megakaryocytes, traditionally known for their role in platelet production, have recently emerged as key players in immune responses during infections. In this study, we explore the involvement of pulmonary megakaryocytes in the response to PA infection, with a specific focus on the mechanistic activation of megakaryocytes via bacterial toxins. We demonstrate that PA infection triggers significant alterations in chemokine signaling and NF-κB pathways in pulmonary megakaryocytes, leading to the upregulation of pro-inflammatory cytokines and chemotactic factors. Activated pulmonary megakaryocytes effectively promote the recruitment of phagocytes, enhancing the host’s resistance to PA infection. we identify PcrV as a key NF-κB activator in megakaryocytes, evidenced by elevated expression of inflammatory markers and chemokines in response to PcrV. Inhibition of NF-κB signaling dampens this inflammatory response, confirming its critical role in megakaryocyte. Together, these findings provide novel insights into the immune functions of megakaryocytes as active modulators during bacterial infections. This work underscores the intricate crosstalk between megakaryocytes and phagocytes and suggests that targeting megakaryocyte activity may represent a promising strategy for controlling bacterial pneumonia.

**Supplementary Information:**

The online version contains supplementary material available at https://doi.org/10.1186/s12964-026-03072-y.

## Background

Megakaryocytes (MKs) are primarily found in the bone marrow and generate thousands of platelets over their lifespan, which involved in coagulation, inflammation, angiogenesis, and immune regulation [1–4]. The roles of platelets in infection and inflammation have guided an interest to the study of extramedullary thrombopoiesis and MKs have been increasingly reported within the spleen and lung. Recent studies have suggested that MKs also play an important regulatory role in asthma, viral infections and pulmonary hemorrhage [5, 6], although the specific regulatory mechanisms remain unclear. For example, MKs are involved in viral infections. In SARS-CoV-2 infection, the transcription levels of CD147 and KREMEN1 in MKs and platelets are upregulated, while NRP1 is downregulated, indicating that SARS-CoV-2 infection can alter the gene transcription of both platelets and MKs [7]. Further studies have shown that SARS-CoV-2 infection significantly activates the NF-kB signaling pathway in MKs, leading to high expression of cytokines such as IL-1β, IL-6, and pattern recognition receptors like TLR2 and TLR4, suggesting that MKs may play an immunoregulatory role during viral infections [8, 9].

MKs are the cell source of circulating platelets, and until recently were typically only considered bone marrow-resident cells. However, accumulating evidence in recent years has begun to highlight the broader physiological functions of MKs in immune modulation and inflammation. Platelet-producing MKs also reside in the lung, and lung MKs express greater levels of immune molecules compared with bone marrow MKs [10–12]. Lung MKs had gene expression patterns that are similar to antigen-presenting cells. The immune phenotype of MKs was plastic and driven by the tissue immune environment. MKs internalized and processed both antigenic proteins and bacterial pathogens. Furthermore, MKs induced CD4 + T cell activation in an MHC II-dependent manner [11, 13, 14]. Beyond these emerging immune roles, the classical functional programs of MKs, including thrombopoiesis and the expression of structural or adhesion molecules, undergo significant remodeling upon pathogen exposure. Evidence from viral infections, such as SARS-CoV-2, influenza and dengue, demonstrates that pathogenic challenges can alter the expression and functionality of key integrins, notably αIIb (CD41), illustrating that the traditional and immunological functions of MKs are deeply interconnected rather than independent [7, 15, 16]. These observations suggest that the immune activation of MKs occurs in parallel with shifts in their conventional functional programs, reflecting a coordinated phenotypic plasticity that integrates host defense with homeostatic maintenance during infection. Notably, transcriptional profiling demonstrated that lung MKs have a unique signature distinct from their hematopoietic counterparts, with lung MKs displaying enrichment for inflammatory response and chemokine mediated signaling activation [17]. This expanding understanding has led to a growing interest in the role of MKs in immune responses, suggesting they may contribute to processes beyond thrombopoiesis, including host immune defense.

The lungs, as a vital organ and the primary interface with the external environment, are constantly exposed to various pathogens, including bacteria, viruses, and fungi [18, 19]. Among these, PA is one of the most common pathogens responsible for lung infections. As the aging of the population becomes more pronounced, the incidence of hospital-acquired pulmonary infections caused by PA has been steadily increasing. Due to the ability to easily colonize, mutate, and develop multidrug resistance, PA diverse pathogenic mechanisms and resistance make it difficult for the immune system to fully clear the infection or for antibiotics to achieve complete cure, resulting in persistent infections [20, 21]. Effective defense against PA infection in the lungs requires the orchestrated activation of multiple immune cell types. Neutrophils and monocytes, migrate to the lung by cytokines and chemokines released by pulmonary macrophages [22], and release cytokines like IL-1, IL-6, and TNF-α to coordinate the immune response. They control the infection through killing PA via phagocytosing and releasing antimicrobial proteins, reactive oxygen species (ROS), and enzymes [23–25]. Dendritic cells serve as key intermediaries between the innate and adaptive immune responses. They capture and process bacterial antigens at the infection site before migrating to lymph nodes, where they present the processed antigens to naïve T cells, which activates T cells and initiates a targeted adaptive immune response [26]. While these immune cells are extensively studied in the context of lung infections, the role of pulmonary MKs in the response to such infections remains poorly understood.

Studies have shown that MKs can migrate from the bone marrow to peripheral tissues, such as the lungs, in response to inflammatory signals [12]. MKs may directly interact with other immune cells, including macrophages and neutrophils, through cytokine secretion [27, 28]. Moreover, MKs might contribute to the early immune response by activating complement cascades or presenting antigens to T cells, functioning similarly to conventional antigen-presenting cells such as dendritic cells [11, 29]. However, the role of MKs in PA infection and the underlying mechanisms remain poorly understood, highlighting the necessity for further investigation into the involvement of lung MKs in the immune response to PA. In this study, a mouse model of acute pulmonary infection was utilized to examine the recruitment, activation, and functional dynamics of MKs within the lung. Our findings revealed that MKs are rapidly recruited to the lungs during the early stages of PA infection, where they become activated and secrete pro-inflammatory cytokines, including IL-1, IL-6, and TNF-α. Furthermore, lung MKs interact with monocytes and neutrophils, potentially modulating the local immune response. Notably, these MKs contribute to infection resolution by promoting pathogen clearance, underscoring their critical role in host defense against pulmonary PA infection. These findings offer novel insights into the previously underappreciated role of MKs in pulmonary immunity, emphasizing their significance not only in thrombopoiesis but also as active modulators of immune responses during infection.

## Methods

### Bacterial strains, cell lines, and animals

The PA PAO1 strain and its derivative mutants were cultured in Luria-Bertani (LB) broth at 37 °C with shaking at 200 rpm. Escherichia coli strain JM109 and BL21 (DE3), employed for protein expression, were grown at 30 °C in LB broth. Mutant strains were constructed using the lambda red system as described previously [30]. Successfully recombinant cells with positive colonies were further confirmed by PCR and DNA sequencing. Antibiotics were used at the following concentrations: ampicillin (100 µg/ml) for E. coli strains, and carbenicillin (300 µg/ml) and Tetracycline (50 µg/ml) for PA. Acetamide plates were used exclusively for growth of PA. All strains used in this study are listed in Supplementary Table 1.

Primary cells sorted by flow cytometry were maintained in Dulbecco’s Modified Eagle’s Medium (DMEM, HyClone), supplemented with 10% fetal bovine serum (FBS, HyClone) and incubated under 5% CO2 at 37 °C.

BALB/c mice, 8 weeks old with body weights ranging from 20 to 25 g, were obtained from the Animal Center of the Third Military Medical University. The PF4-Cre; iDTR (inducible diphtheria toxin receptor) mouse, which was used in megakaryocyte knockout experiments, was kindly provided by Prof. Liu of Sun Yat-sen University. C57/BL6 mice normally housed in the Specific Pathogen Free Animal Room in accordance with the Army Medical University and Chinese national laboratory animal care and usage guidelines.

### Expression and purification of PcrV and Ndk protein

The protein expression and purification were performed as described previously with some modifications. The PA PcrV and Ndk genes were cloned into the pQE31 vector (Qiagen, Germany), which facilitates the addition of an N-terminal His₆ tag to the recombinant proteins. The Escherichia coli JM109 strain harboring the expression plasmids was cultured in LB medium supplemented with 100 µg/mL ampicillin until the OD₆₀₀ reached 0.5. Expression was then induced by adding 0.5 mM isopropyl-β-D-thiogalactopyranoside (IPTG), and incubation continued at 37 °C for 4 h. Following induction, the cells were harvested, resuspended in phosphate-buffered saline (PBS), and lysed by sonication. The fusion proteins were subsequently purified via affinity chromatography using a His-Trap HP column (GE Healthcare, Germany). Endotoxin removal from PcrV and Ndk was achieved by treatment with Detoxi-Gel™ endotoxin removal gel (Thermo Fisher Scientific, USA) according to the manufacturer’s protocol.

### Animal experiments

The mouse acute lung infection model was conducted as previously with minor modifications [31]. In brief, overnight cultured bacteria were re-inoculated into fresh LB medium with a starting concentration of OD600nm = 0.01, continue culturing for three hours so that the bacteria are in a log-phase (OD600nm around 0.5). After sterile PBS washing, bacterial suspension is adjusted OD600nm to 0.3, then 8-week-old male C57 mice were anesthetized by intraperitoneal injection of sodium pentobarbital (50 mg/kg) and then inoculated with 50 µl bacterial suspension via tracheotomy. Mice were sacrificed with CO_2_ 20 h post-infection and the right lobe of the lung were shred and digested for flow cytometry analysis, and the left lobe of the lung were removed for pathological HE staining.

The pneumonia model induced by PA toxins were conducted as follow. The lipopolysaccharide (LPS) and pyocyanin of PA were purchased from Sigma, USA. The PcrV and Ndk proteins were expressed and purified in the laboratory. Pyocyanin was dissolved in dimethyl sulfoxide (DMSO) to prepare a stock solution of 2 mg/mL, which was stored at − 20 °C from light, the stock solution was diluted 10-fold with H_2_O before use. The LPS, PcrV, and Ndk were dissolved in PBS) and then stored at − 20 °C. Upon use, they were prepared at the following concentrations: pyocyanin (200 µg/mL), LPS (200 µg/mL), PcrV (500 µg/mL), and Ndk (500 µg/mL). Then 8-week-old male C57 mice were anesthetized by intraperitoneal injection of sodium pentobarbital (50 mg/kg) and then inoculated with 50 µl suspension via intratracheal. For intratracheal diphtheria toxin (DT) administration, *Pf4-Cre; iDTR* mice and their control counterparts were given a single dose of either 50 ng DT or PBS (in a 50 µl volume) via intratracheal inoculation daily for the initial two days. On day 3, MK depletion efficiency was verified. On day 4, mice were challenged with PA via intratracheal inoculation; 20 h post-infection, the mice were sacrificed, and lung tissues were harvested for flow cytometric analysis. Mice were sacrificed with CO_2_ 20 h post-infection and the right lobe of the lung were shred and digested for flow cytometry analysis, and the left lobe of the lung were removed for pathological HE staining.For preliminary estimation of bacterial load of lung tissue, the tissue lysates underwent a 10-fold dilution were spot-like inoculated individually to predict bacterial loads via a pre-test. The properly diluted lysates were plated on acetamide agar plates and the bacterial load were determined by colony-forming unit (CFU) counting.

### Platelet isolation and washing

Donor platelets were isolated from age-matched wild-type C57BL/6 mice. Briefly, whole blood was collected via the inferior vena cava into syringes containing heparin (final concentration 25 U/ml) and supplemented with 10% acid citrate-dextrose (ACD). The blood (500 µl) was diluted with 300 µl Tyrode’s HEPES buffer (TH; 140 mM NaCl, 3 mM KCl, 1 mM MgCl_2_, 16.6 mM NaHCO_3_, 10 mM HEPES, pH 7.4) and centrifuged at 200 g for 6 min. The resulting platelet-rich plasma (PRP) and the upper two-thirds of the erythrocyte fraction were collected and re-centrifuged at 100 g for 6 min to maximize platelet yield. To prevent premature activation during processing, the gathered platelets were pelleted at 1,000 g for 90 s in the presence of 1/25 volume of ACD and 0.5 U/ml apyrase. The final platelet pellet was resuspended in calcium-free TH buffer, and the concentration was adjusted to 1*10^9^ platelets/ml using an automated hematology analyzer.

### Platelet transfusion and evaluation of host defense

Recipient mice (Pf4-Cre mice previously treated with intra-tracheal DT to induce pulmonary MK depletion) were used for the rescue experiments. Two hours prior to PA infection, 1* 10^8^ washed WT platelets (suspended in 100 µl TH buffer) were administered to the experimental group via tail vein injection (i.v.) to restore circulating platelet counts to physiological levels. Control recipient mice received an equal volume of TH buffer (vehicle) alone. Following transfusion, all recipient mice were challenged with PA via intra-tracheal inoculation as previously described. At 20 h post-infection, mice were sacrificed to assess the rescue effect. The recruitment of neutrophils and monocytes was quantified in bronchoalveolar lavage fluid (BALF) via flow cytometry. Additionally, lung tissues were harvested, homogenized, and serially diluted for CFU assays to determine the pulmonary bacterial load.

### Mice survival experiments

The bacterial suspension was prepared as before, adjust concentration to 0.1, 0.3, and 0.5 (OD600nm), then 8-week-old male C57/BL6 mice were anesthetized by intraperitoneal injection of sodium pentobarbital (50 mg/kg) and then inoculated with 50 µl bacterial suspension via tracheotomy. The survival rate of the mice was monitored at 5-hour intervals. Ultimately, the intermediate dose (OD600nm = 0.3) that caused significant inflammatory infiltration and hemorrhage in the lungs without killing the mice within 20 h was selected for survival experiments.

### Tissue pre-treatment for flow cytometry

For lung tissue, the flow cytometry analysis was conducted as previously with a few modifications [11]. Briefly, 20 h post PA infection, the right lobe of the lung was shred and digested in 1mL digestive buffer (1 mg/mL Collagenase D, 0.1 mg/mL DNaseI, 2mM L-glutamine, 2% FBS, 25mM HEPES resolved in Hank’s balanced salt solution) for 30 min at 37 ℃. Then the digested tissue was homogenized with syringe shanks prior to filtered through a 70 μm strainer. Erythrocytes present in the homogenates were lysed using a dedicated lysis buffer, followed by two washes. Any remaining red blood cells were lysed using ACK lysis buffer (Gibco) for a duration of 2 min. Cells were washed with PBS and resuspended in PBS containing 1%FBS for further stained with fluorochrome-conjugated antibodies for 60 min at 4℃. Samples were analyzed using a flow cytometer (BD Fortesa), and data were analyzed using FlowJo 10.5.3.

### Flow cytometry and gating strategy

For pulmonary leukocyte and MK analysis, cells were first gated on a forward scatter area (FSC-A) versus side scatter area (SSC-A) plot to exclude debris. To strictly eliminate cell aggregates and doublet events, a critical step in a platelet-rich inflammatory context—consecutive singlet gating was performed using FSC-A versus forward scatter height (FSC-H).

The sorting and analytical gates for pulmonary MKs were designed based on cell size and lineage-specific markers. Given that the forward scatter parameter correlates with cell diameter, high forward scatter (FSC^hi^>150) was utilized to distinguish large MKs from the majority of standard immune populations. The MK-specific marker CD41 was subsequently employed to exclude non-specific immune cell interference. Briefly, FSC^hi^CD41 + cells were identified as MKs, within which specific subsets were further gated as CD48 + MKs (FSC^hi^CD41 + CD48+), CD11c + MKs (FSC^hi^CD41 + CD11c+), and CXCR4 + MKs (FSC^hi^CD41 + CXCR4+). The comprehensive gating hierarchy for MKs is illustrated in Supplementary Fig. 2A.

Other major leukocyte subpopulations were analyzed following the sequential gating hierarchy indicated by the routing arrows in Supplementary Fig. 2B, using the following criteria: Myeloid cells (CD45 + CD11b+), Neutrophils (CD45 + CD11b+F4/80 − CD86+), Macrophages (CD45 + CD11b+F4/80 + CD86−), and Monocytes (CD45 + CD11b+F4/80 − CD86−Ly6C+). All antibodies utilized in this study are detailed in Supplementary Table 2.

### Ploidy analysis of lung MKs

To determine the ploidy profile of lung MKs and exclude potential artifacts from platelet-immune cell complexes, DNA content analysis was performed. Briefly, single-cell suspensions from lung tissues were stained with surface markers (CD41 + and gating for large cells via FSC). Intracellular DNA was stained with DAPI containing RNase A for 30 min at room temperature in the dark. Flow cytometry data were acquired and analyzed using FlowJo software to quantify the distribution of cellular DNA content (2 N, 4 N, 8 N, etc.).

### Flow sorting for bone marrow cells

Bone marrow cells were collected from the femurs of C57/BL6 mice. Murine monocytes, neutrophils and MKs were isolated using the fluorescence activated cell sorting (FACS) technique. Briefly, femurs were collected from four mice under sterile conditions and the ends were decapitated, and then transferred to a dish containing 10 mL of PBS. Using a 1 mL syringe, the cells in bone marrow were thoroughly washed out and transferred to a 15 mL centrifuge tube. The cells were rinsed with PBS and resuspended in 2 mL of RBC lysis buffer and incubated for 3 min to lyse the red blood cells and stopped by adding 3 mL of PBS. After filtering through sterile filter mesh, the cell suspension was rinsed with PBS. The pellet was resuspended in 6 mL PBS (PBS supplemented with 5% FBS were used in the later step). Take 2 mL of the cell suspension for phagocyte sorting and incubate with 5 µL of each specific antibody corresponding to the markers, and 4 mL of the cell suspension for MKs sorting incubate with 20 µL of each specific antibody corresponding to the markers. The staining were incubated for 60 min at 4 °C in the dark. After washing with PBS, cells were resuspended in an equal volume of PBS, and filtered through a cell strainer prior sorting. The sorted cells were collected directly in DMEM containing 10% FBS, and counted in a cytometer. Adjusted the cell suspensions to 10^5^ cells/mL and cultured at 37 °C in a 5% CO2 environment. The following fluorescent antibodies were used for staining and sorting: (1) For phagocytic cells: CD45-APCcy7, CD11b-Percpcy5.5, LY6G-FITC, LY6C-BV421, F4/80-APC and Zombie-PE; (2) for MKs: CD41-APC, CD48-APCcy7 and Zombie-PE.

### Chemotaxis assay

Cells were cultured in RPMI-1640 medium supplemented with 10% FBS, 1% pen-strep, at 37 °C in a 5% CO2 environment. For cell chemotaxis assay, the Trans-well was carried out. Neutrophils and monocytes were seeded in the upper chamber in which the upper and lower cultures were separated by a polycarbonate membrane (8 μm pore size), and due to the permeability of the polycarbonate membrane, components secreted by the MKs in the lower culture medium could influence cell movement in the upper chamber. Briefly, the flow-sorted MKs were collected by centrifugation at 500 g for 5 min, and the cells were resuspended with 10% FBS medium and the density was adjusted to 1*10^5^ /mL; the MKs were homogenously divided into the lower chamber according to 0.5 mL/well, and four subgroups were set up (blank control group, PcrV group, DMSO+PcrV group, QNE+ PcrV group), each with 3 replicates. Firstly, the MKs of QNE+PcrV group was treated with 50nM QNE for 30 min, and then PcrV (10 µg/ml) was added to groups 2,3,4 for 1 h. After that, the density of flow-sorted neutrophils and monocytes was adjusted to 1*10^6^/ml using serum-free medium, and then the cell suspensions were mixed at a ratio of 3:1, according to 200 µL/well. The cell suspension was homogenized into the upper chamber and cultured for 12–48 h (depending on the migration ability of the cells). Cells were fixed with 4% paraformaldehyde and then stained with 0.2% crystal violet to detect the number of cells passing through the polycarbonate membrane, and the counting results made into statistical graphs.

### In vitro cell experiment

Bone marrow MKs were sorted by flow cytometry and centrifuged at 500 g for 5 minutes to collect the cells. The cells were then resuspended in medium containing 10% FBS and 1% pen-strep, and the cell density was adjusted to 5*10^4^ cells/mL. The cell suspension was evenly distributed into a 6-well plate, 2 mL per well, with four groups established (PBS group, PcrV group, PcrV+DMSO group, PcrV + QNE group), each with three replicates. Next, 50 nM QNE was added to the PcrV + QNE group, and the cells were incubated at 37 °C for 30 min. Then, PcrV (10 µg/mL) was added to groups 2, 3, and 4, and the cells were cultured at 37 °C for 3 h. After incubation, the cells were collected by centrifugation, washed once with PBS, and then resuspended in 800 µL of TRIzol. The resulting product was stored at -80 °C or immediately used for RNA extraction. For the neutrophil and monocyte in vitro stimulation experiments, the procedure was the same, with a slight adjustment in cell density. The flow-sorted cells were centrifuged at 500 g for 5 minutes, resuspended in medium containing 10% FBS and 1% antibiotics, and the cell density was adjusted to 1*10^5^ cells/mL.

### RNA isolation and RT-qPCR

The RNA isolation was performed using TRIZOL reagent as previously [32] with a little modification. Briefly, thaw the samples which containing 0.8 mL TRIzol on ice. Add 200 µL of chloroform into the samples and mix thoroughly, allow the mixture to stand for phase separation. Centrifuge at 12,000 rpm for 15 min. Transfer the supernatant to a new tube and add an equal volume of isopropanol. Then, add 2 µL of glycogen solution (20 mg/mL) and 1/10th the volume of 2 M NaCl solution. Mix well and let stand for 10 min. Centrifuge at 12,000 rpm for 10 min and discard the supernatant. Finally, wash the pellet once with 75% ethanol. The precipitate is dissolved in 10 µl of DEPC H2O and the product is ready for subsequent PCR analysis.

For RT-qPCR, 1 ug total RNA was reverse transcribed to cDNA for further RT-qPCR analysis using SYBR Green. RT-qPCR was performed on an ABI 7500 RT-PCR system. The gene Gapdh served as endogenous control. Changes in gene expression were calculated using the ΔΔCt method, based on the mean change in RT-qPCR cycle threshold (ΔCt). Primers were designed using NCBI Primer-BLAST tool, and product melting curves were done to validate the specificity of the primers, with the specific sequences provided in Supplementary Table 3.

### RNA-seq analysis

RNA-seq analysis was performed by Shanghai Biotechnology Corporation (Shanghai, China) as normal. Briefly, after constructing the mice model of acute pneumonia using the PAO1 strain, pulmonary MKs were sorted by flow cytometry for SMART sequencing. Total RNA was extracted following standard procedures, and RNA concentration was measured simultaneously using Qubit 3.0 (Thermo Fisher Scientific, MA, USA) and Nanodrop One (Thermo Fisher Scientific, MA, USA). RNA integrity was accurately assessed using the Agilent 4200 system (Agilent Technologies, Waldbronn, Germany). mRNA sequencing libraries were constructed using the NEB Next^®^ Ultra™ Directional RNA Library Prep Kit (New England Biolabs, MA, USA), following the manufacturer’s protocol. The indexed samples were clustered on a cBot cluster generation system. After clustering, the library was sequenced on the Illumina Novaseq6000 platform, generating 150 bp paired-end reads. Differentially expressed genes were identified using DESeq2 (v1.34.0). Genes with FDR ≤ 0.05 and log2(Fold Change) ≥ 1 were defined as differentially expressed and selected for further investigation. Significantly enriched GO terms were identified using an FDR threshold of ≤ 0.05.

### Statistics and reproducibility

Statistical differences between group means were evaluated using two-tailed Student’s t-test or the Mann Whitney U test, based on the specific scenario. Statistical analyses were conducted using Prism GraphPad 8. A P-value < 0.05 was considered significant. The data points across figures represent the mean ± SD of values as noted. Significance levels were denoted as: *P ≤ 0.05; **P ≤ 0.01; ***P ≤ 0.001; with “ns” indicating not significant (*P* > 0.05). All experiments were replicated for reproducibility. The precise number (n) of samples in animal and in vitro studies were detailed in the accompanying figures and their legends.

### Data availability

Data supporting the findings of this manuscript could be obtained from the corresponding author upon reasonable request. The RNA-seq data is uploaded to SRA with an accession number: GSE295916.

## Results

### PA challenge induces a transient accumulation of pulmonary MKs and immune-related phenotypic shifts

**Fig. 1:**
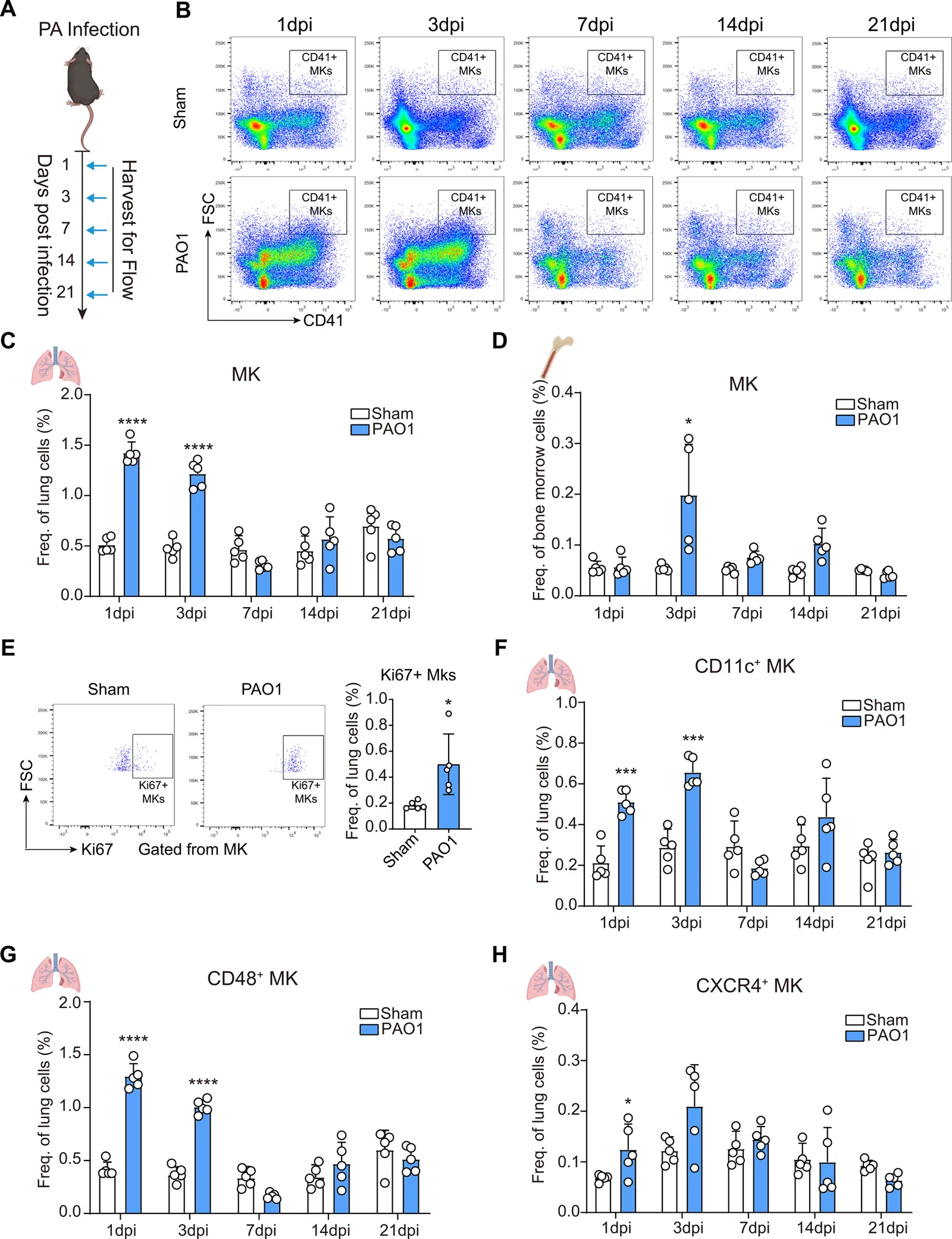
The pulmonary MKs were expanded under PA induced acute pneumonia. **A** The schematic shows the model of mouse acute pneumonia induced by PA infection, the lung tissue and bone marrow were harvested on days1, 3, 7, 14 and 21 post-infections to investigate the potential role of MKs under PA infection. **B** Representative FACS plot of pulmonary MKs in the mice from the PA induced acute pneumonia group compared to the control group. **C** Statistical analysis showing the ratio of pulmonary MKs in the mice from the PA induced acute pneumonia group compared to the control group (*n* = 5). **D** Statistical analysis showing the ratio of bone marrow MKs in the mice from the PA induced acute pneumonia group compared to the control group (*n* = 5). **E** Representative FACS plot (left) and cumulative % (right) of cycling pulmonary MKs (CD41 + Ki67+) in control and PA-infected mice (*n* = 5). (**F**, **G** and **H**) Statistical analysis showing the cumulative % of CD48 + MKs (**F**), CD11c + MKs (**G**), CXCR4 + MKs (**H**) in the mice from the PA induced acute pneumonia group compared to the control group (*n* = 5). The data shown were mean ± SD. Statistical significance by a two-tailed unpaired t-test is indicated: *P ≤ 0.05; **P ≤ 0.01; ***P ≤ 0.001; ns, not significant (*P* > 0.05)

Studies shown that pulmonary MKs can perform thrombopoiesis and express molecules like toll-like receptors and MHC II, enabling antigen presentation and leukocyte activation [7, 11, 12]. We propose that MKs in the lung play a key role in clearing inhaled PA. Using a mouse model of acute pulmonary infection, we analyzed lung tissue and bone marrow at days 1, 3, 7, 14, and 21 post-infections (Fig. 1A). Photographs of lung showed significant hemorrhage in PA infected mice on days 1 and 3 compared to control group (Supplementary Fig. 1A). CFU counts showed that high levels of PA were detected in lung tissue during the first week after PA infection (Supplementary Fig. 1B). Flow analysis of lung tissue showed that neutrophils, monocytes, macrophages, dendritic cells were significantly elevated on days 1 and 3 post PA infection (Supplementary Fig. 1C-G). The results confirmed that a mouse model of acute lung infection was established, providing a solid foundation for further investigation into the role of MKs in the immune response to PA infection.

Flow analysis of lung tissue showed a significantly higher proportion of MKs in the PA-infected group compared to the control group on days 1 and 3 post-infection (Fig. 1A and B), while there was no significant difference in the proportion of MKs in the lungs on days 7, 14, and 21 post-infections between two groups (Fig. 1B). The statistical results are shown in Fig. 1C. The alterations in MKs within the bone marrow did not entirely align with those observed in the lungs. Specifically, the proportion of MKs in the bone marrow did not show a significant increase when compared to the control group on the first day post-infection (Fig. 1D). On the third day post-infection, the proportion of MKs in the bone marrow began to rise. However, by days 7, 14, and 21, the proportion of MKs returned to baseline levels (Fig. 1D). Of note, the pulmonary MKs in active cell cycle state in the infected group was significantly higher than that in the control group after 1 day of infection (Fig. 1E).

To evaluate the phenotypic remodeling of pulmonary MKs following infection, we analyzed the expression profiles of immune-related surface molecules, including CD11c, CD48, and the chemokine receptor CXCR4. Previous studies have indicated that lung-resident MKs can express higher levels of immune-associated molecules like CD11c compared to their bone marrow counterparts [11], while CD48 serves as a co-stimulatory molecule traditionally involved in immune cell activation [13, 33]. Flow cytometric analysis revealed that the proportions of MKs expressing CD11c and CD48 significantly increased during the early stages of infection (days 1 and 3 post-infection) before returning to baseline levels in the later stage (Fig. 1F, G). In contrast, the fraction of CXCR4-positive MKs displayed only a transient, slight increase on day 1 (Fig. 1H). These results indicate that PA infection predominantly expands the fraction of MKs bearing reactive immune-related markers. We note that these surface molecules serve as descriptive markers reflecting a responsive, pro-inflammatory state of pulmonary MKs under infectious stress, rather than defining distinct, functionally hardwired anatomical sub-lineages. Collectively, these phenotypic shifts suggest that pulmonary MKs likely participate in modulating the local immune response and shaping the inflammatory milieu during PA infection.

### MKs depletion compromises host defense against PA

**Fig. 2:**
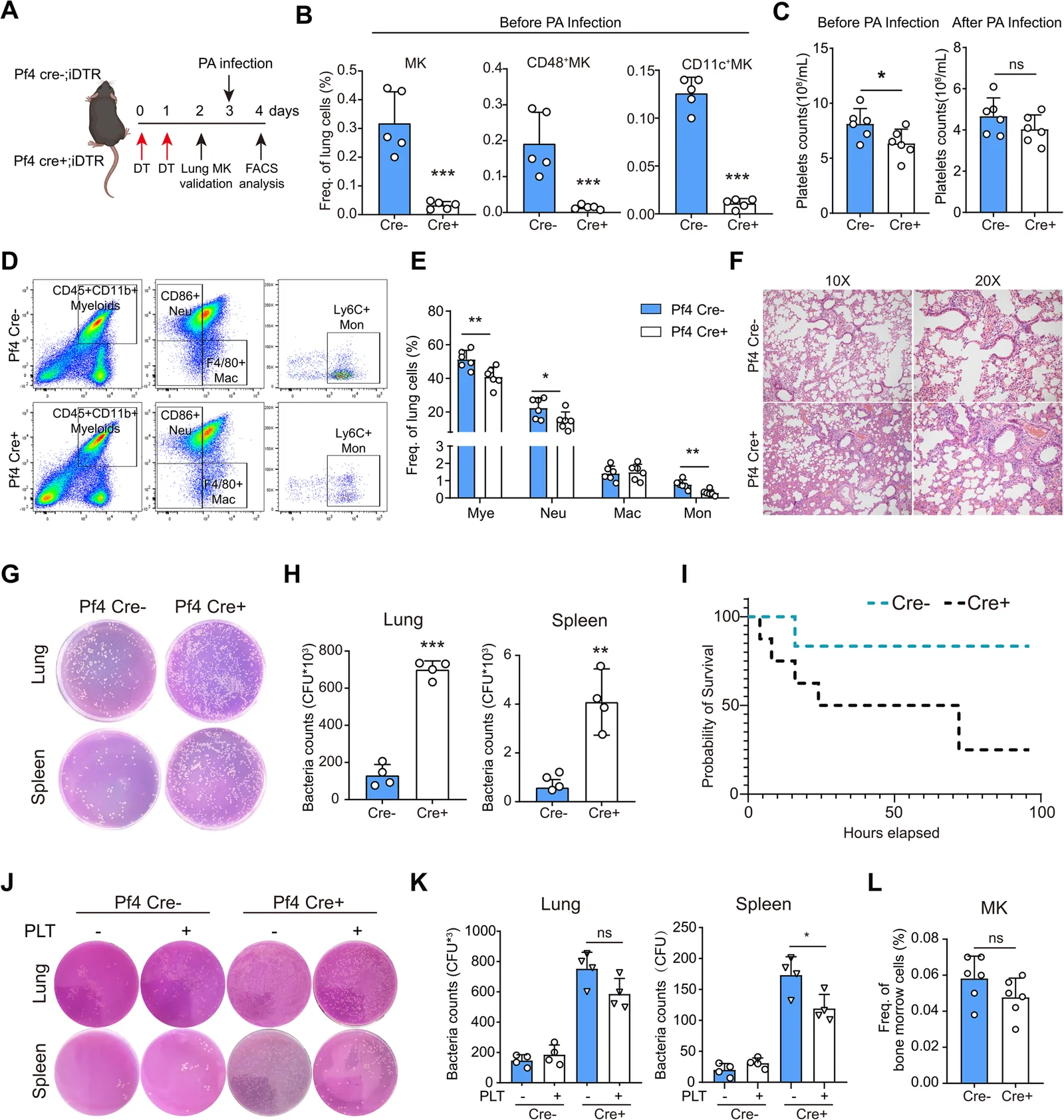
Pulmonary MKs enhance host defense against PA infection. **A** The schematic shows the deletion procedure of lung MKs and the following PA infection procedure in Pf4-cre mice. **B** FACS analysis shows the proportion of pulmonary MKs and the CD48 + or CD11c + MKs in Pf4-cre mice following DT induction prior to *P.a* infection compared to control mice (*n* = 5). **C** Histogram shows circulating platelet counts in DT-induced MK-deletion mice before and after PA infection (*n* = 6). **D** Representative FACS plot of pulmonary phagocytes from Pf4-cre mice following PA infection compared to the control mice. **E** Statistical analysis showing the ratio of pulmonary phagocytes from Pf4-cre mice following PA infection compared to the control mice (Myeloid: Mye; Neutrophils: Neu; Macrophages: Mac; Monocytes: Mon) (*n* = 6). **F** H&E staining of lung tissue sections shows the pathological inflammation in Pf4-Cre mice following PA infection, compared to control mice, magnification is marked above the image. **G** Photographs of CFU counts shows the bacterial load of lung and spleen of Pf4-Cre mice after PA infection compared to control mice. **H** Statistical analysis of the CFU counts shows the bacterial load of lung and spleen of Pf4-Cre mice after PA infection compared to control mice (*n* = 4). **I** After MK deletion, the survival assay shows the survival ratio of Pf4-cre mice after PA infection compared to control mice (*n* = 10). **J** Photographs of CFU counts shows lung and spleen bacterial load in DT-induced MK-deletion mice following platelet transfusion experiments. **K** Statistical analysis of the CFU counts shows lung bacterial load in DT-induced MK-deletion mice following platelet transfusion experiments (*n* = 4). **L** FACS analysis shows the proportion of bone marrow MKs in Pf4-cre mice following DT induction compared to control mice (*n* = 6). The data shown were mean ± SD. Statistical significance by a two-tailed unpaired t-test is indicated: *P ≤ 0.05; **P ≤ 0.01; ***P ≤ 0.001; ns, not significant (*P* > 0.05)

The accumalation of pulmonary MKs during PA infection reflects their infection-associated pro-inflammatory state, thereby emphasizing their potential as reactive players in acute lung injury. Ploidy analysis confirms the identity of lung MKs (Supplementary Fig. 2C). Notably, infectious stress significantly decreased high-ploidy MK enrichment, instead driving an increase in the proportion of 2–4 N MKs compared to controls. This elevated proportion of low-ploidy MKs indicates a state of heightened immune responsiveness, which is consistent with previous reports [11]. However, it remains unclear whether the role of MKs in response to PA infection is beneficial or detrimental. To investigate whether pulmonary MKs are beneficial to the host defense against acute PA infection, we used Pf4-Cre; iDTR mice combined with intratracheal administration of DT to specifically induce MK depletion within the lung (Fig. 2A). Before PA infection, flow cytometry analysis revealed a significant reduction in total lung MKs, including the CD48 + and CD11c + MKs, in Pf4-Cre mice following DT-induced depletion compared to controls (Fig. 2B). While the circulating platelet counts before and after PA infection showed only a slight decrease in Cre+ mice compared with Cre- mice, and far from being completely depleted in either condition (Fig. 2C). To evaluate inflammatory infiltration following PA infection in the two genotypes, flow cytometric analysis was performed, revealing a lower percentage of myeloid cells in the lung tissue of Pf4-Cre mice compared to controls. Specifically, the percentage of neutrophils and monocytes, but not macrophages, were lower in Pf4-Cre mice than control mice after PA infection (Fig. 2D, E). Histological staining revealed that the pulmonary lesions had become more severe and were accompanied by an increased tendency to bleed, which could be associated with a deficiency of MKs in Pf4-Cre mice compared to the control group (Fig. 2F). However, the bacterial load of lung tissue showed that the CFU counts of Pf4-Cre mice was significantly higher than the control mice (Fig. 2G, H). Given that the severe bacterial pneumonia often results in bloodstream infections, the spleen bacterial load was assessed to reflect the blood bacterial load. The CFU counts revealed that the bacterial load in spleen of Pf4-Cre mice were significantly higher than those in control mice (Fig. 2G, H), which indicated that megakaryocyte-depleted mice exhibited susceptibility to PA infection and enhanced the risk of bloodstream infections. Furthermore, survival analysis showed that Pf4-Cre mice had a higher mortality rate after PA infection compared to control mice (Fig. 2I), the higher mortality rate may be due to a faster proliferation of PA in the lung and a worse bloodstream infection in Pf4-Cre mice compared to the control mice.

To determine whether the antibacterial defect of MK-depleted mice stems solely from thrombocytopenia, we performed platelet transfusion. Although platelet replenishment lowered the lung and spleen bacterial load in Cre+ mice, their bacterial burden remained higher than that of Cre- controls (Fig. 2J, K). This partial rescue demonstrates that platelets are protective but insufficient to fully restore immunity. Concurrently, no significant difference in bone marrow MK counts was observed upon DT-induced MK-depletion between Cre- or Cre+ groups (Fig. 2L). This suggests that MKs in the lung play a protective role in the host’s defense against PA infection, potentially through their involvement in immune cell activation, cytokine production, and the modulation of local inflammation.

### Pulmonary MKs exhibit phagocytosis and inflammatory responses upon PA infection

**Fig. 3:**
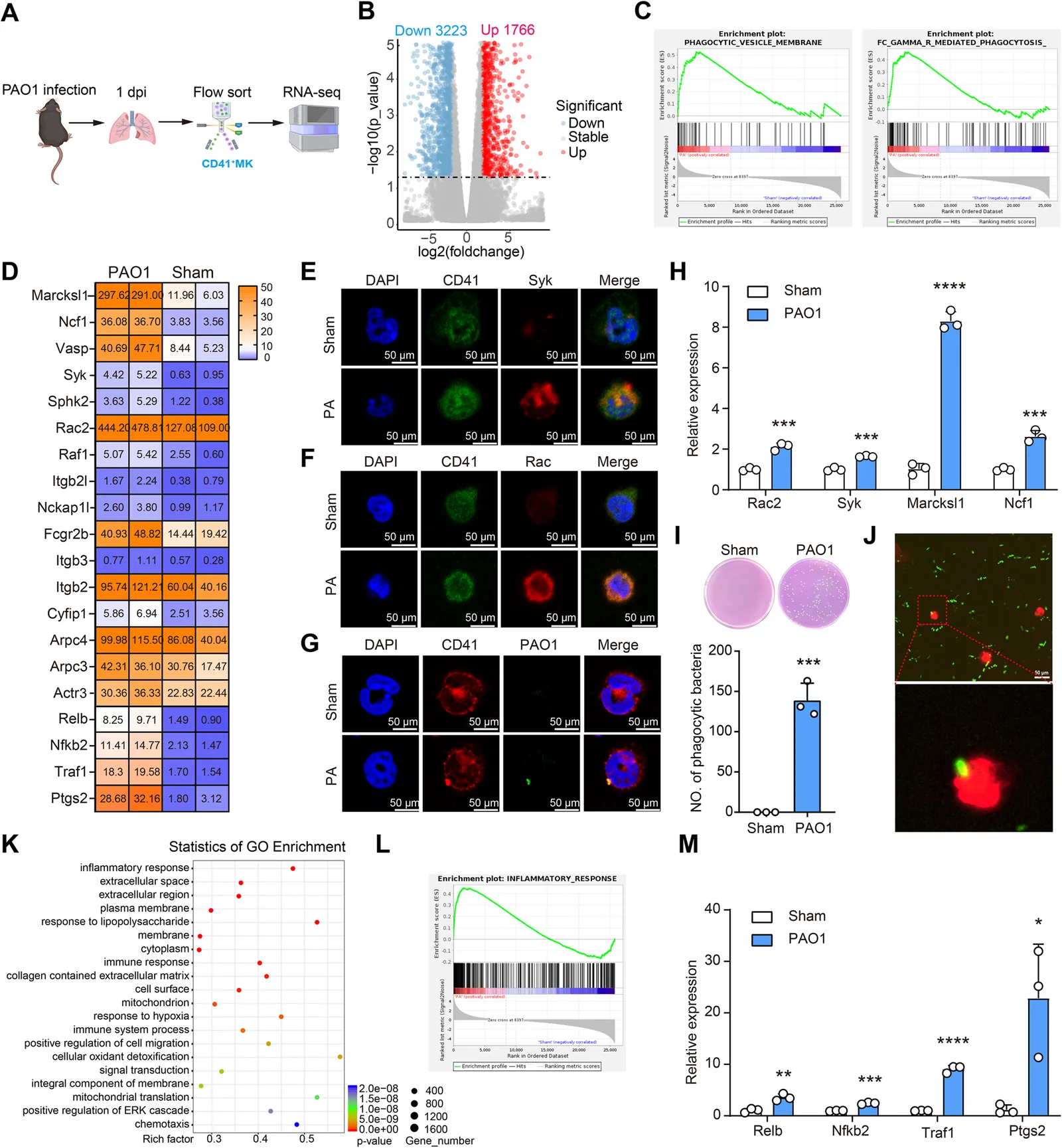
The phagocytic signaling and inframammary response of pulmonary MKs were activated under PA infection. **A** The schematic shows the flow sorting and RNA-Seq procedure for pulmonary MKs, the biological insights illustrated in **B**, **C**, **D**, **K**, **L** were analyzed based entirely on this RNA-seq dataset. **B** The Volcano plot of RNA-seq data shows the significantly up- and down-regulated genes in PA infected mice compared to control group, which have been colored in red (up) and blue (down) respectively (*n* = 2). **C** GSEA analysis shows the activation of phagocytic and FCγ receptor signaling pathway in pulmonary MKs of PA infected mice compared to the control group. **D** The heatmap depicted the expression of phagocytosis related genes and inflammatory response markers in pulmonary MKs of PA infected mice compared to control group. up- and Down- regulated genes are colored by orange and blue respectively (*n* = 2). **E**, **F** and **G** Representative fluorescence confocal image shows the protein expression of Syk, Rac and the swallowed bacteria in pulmonary MKs of PA infected mice (24 h post infection) compared to the control group. **H** RT-qPCR analysis shows the expression of phagocytosis marker genes (Rac2, Syk, Marcksl1, Ncf1) in pulmonary MKs of PA infected mice (24 h post infection) compared to the control group (*n* = 3). **I** Photographs of CFU counts (upper) and the statistical analysis (lower) shows the bacterial load in pulmonary MKs of PA infected mice (24 h post infection) compared to the control group (*n* = 3). **J** Representative live-cell imaging snapshots of FACS-purified pulmonary MKs engulfing PA in vitro. **K** The statistics of GO enrichment shows major changes in biological process in pulmonary MKs of PA infected mice compared to the control group. **L** GSEA analysis shows the activation of inflammatory response pathway in pulmonary MKs of PA infected mice compared to the control group. **M** RT-qPCR analysis shows the expression of marker genes (Relb, Nfkb2, Traf1, Ptgs2) involved in inflammatory response signaling in pulmonary MKs of PA-infected mice compared to the control group (*n* = 3). The data shown were mean ± SD. Statistical significance by a two-tailed unpaired t-test is indicated: *P ≤ 0.05; **P ≤ 0.01; ***P ≤ 0.001; ns, not significant (*P* > 0.05)

Given that pulmonary MKs contribute to host defense against PA infection in the lung, we aimed to further investigate the underlying mechanisms by which cells contribute to the coordination of the host’s immune response in clearing PA from the pulmonary system. Specifically, we sought to elucidate how pulmonary MKs interact with other immune cells, modulate inflammation response for effective bacterial clearance. The pulmonary MKs were sorted specifically by FACS following PA infection and subsequently utilized Smart-Seq technology to analyze the transcriptomic changes (Fig. 3A). Smart-Seq analysis revealed that, compared to control group, the expression of 1,766 genes was significantly upregulated while 3,223 genes were significantly downregulated in the pulmonary MKs following PA infection (FDR ≤ 0.05 and log2(fold change) ≥ 1) (Fig. 3B). Furthermore, Gene Set Enrichment Analysis (GSEA) revealed that the phagocytosis and FCγ receptor signaling pathways were significantly activated in the pulmonary MKs following PA infection (Fig. 3C), and the heatmap further illustrated the changes in phagocytosis and FCγ receptor signaling pathways (Fig. 3D). Immunofluorescence staining demonstrated that the levels of Syk and Rac proteins were elevated in PA-infected mice compared to the controls (Fig. 3E, F). Immunofluorescence staining confirmed the intracellular presence of PA in flow-sorted MKs, a finding further supported by live-cell imaging that directly visualized the capture and phagocytosis of PA by MKs (Fig. 3G, J). The dynamic process of MKs capturing and phagocytosing PA was showed in Supplementary Video 1. RT-qPCR verified that four key markers (*Rac2*,* Syk*,* Marcksl1*,* Ncf1*) in the phagocytosis pathway were significantly increased in the MKs following PA infection compared to the control group (Fig. 3H). Furthermore, the pulmonary MKs were isolated specifically for CFU assays, data revealed a substantial burden of PA colonies in the infected group (Fig. 3I).

GO enrichment analysis showed numerous signaling related to the inflammatory and immune response were activated in MKs following PA infection (Fig. 3K). GSEA further verified the activation of inflammatory response signaling in MKs following PA infection (Fig. 3L). The heatmap highlighted distinct expression shifts in several inflammatory markers (*Relb*,* Nfkb2*,* Traf1*, and *Ptgs2*) (Fig. 3D), a trend further validated by RT-qPCR analysis (Fig. 3M). In summary, our findings demonstrate that lung megakaryocytes possess the capacity to uptake and sense bacteria. This distinct feature strongly suggests that they may function as local ‘immune sentinels’ to orchestrate and initiate early inflammatory and host defense responses during respiratory infections.

### Pulmonary MKs orchestrate pro-inflammatory and chemotactic responses via NF-κB activation upon PA infection

**Fig. 4:**
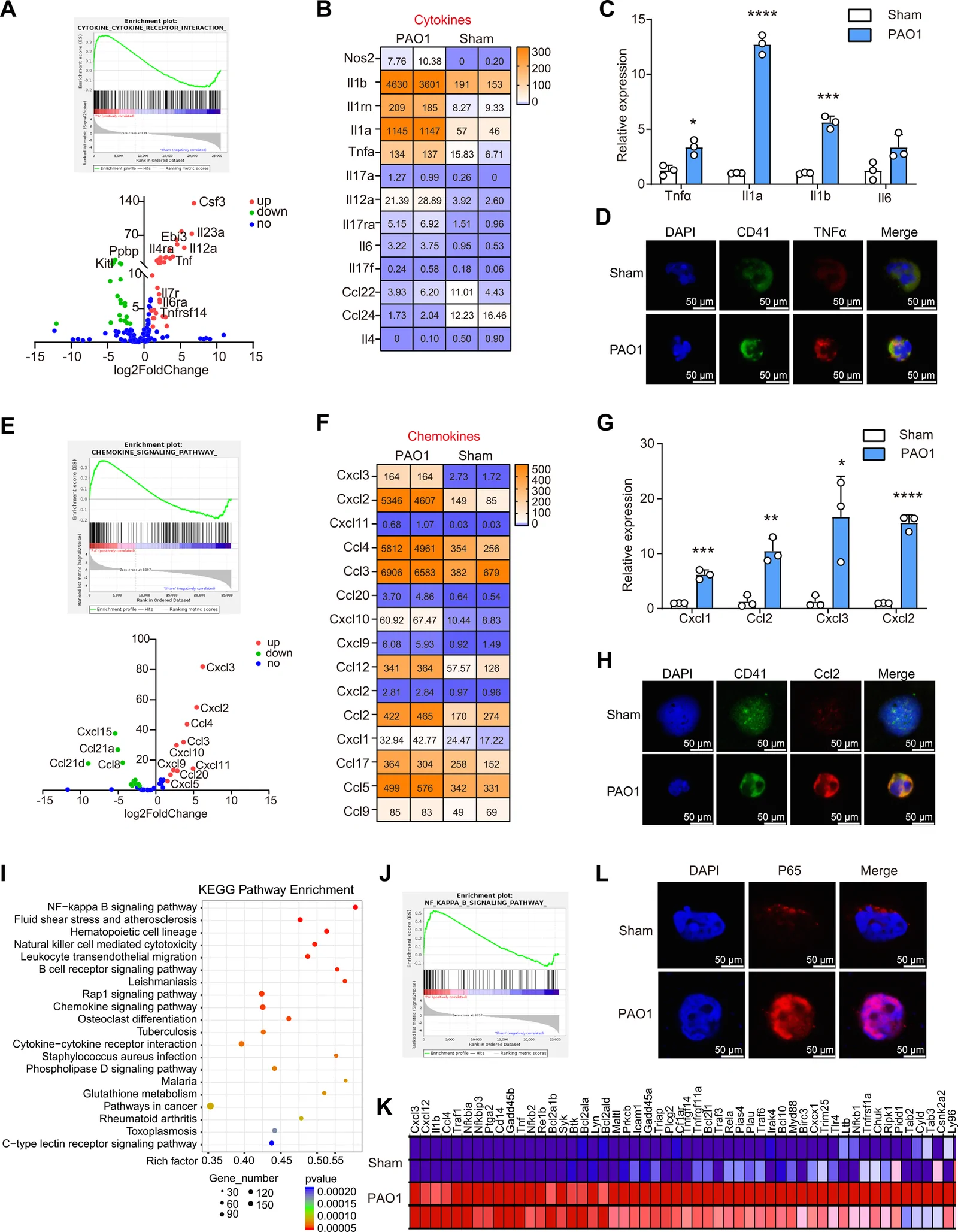
The NF-κb signaling was activated after PA infection in pulmonary MKs. The RNA-seq data presented in Fig. 3A were subjected to deeper analysis, and the biological insights illustrated in **A**, **B**, **E**, **F**, **I**, **J**, **K** were analyzed based entirely on this RNA-seq dataset. **A** and **B** The GSEA analysis and the heatmap shows the activation of cytokines signaling pathway in pulmonary MKs of PA infected mice compared to the control group. The Volcano plot in A (lower) shows the significantly up- and down-regulated cytokines in PA infected MKs. **C** RT-qPCR analysis shows the expression of four key pro-inflammatory cytokines (*Tnfα*,* Il1α*,* Il1β*,* Il6*) in pulmonary MKs of PA-infected mice, compared to the control group (*n* = 3). **D** Representative fluorescence confocal image shows the protein expression of TNFα in pulmonary MKs of PA infected mice compared to the control group. **E** and **F** The GSEA analysis and the heatmap shows the activation of chemokines signaling pathway in pulmonary MKs of PA infected mice compared to the control group. The Volcano plot in **E** (lower) shows the significantly up- and down-regulated chemokines in PA infected MKs. **G** RT-qPCR analysis shows the expression of four key pro-inflammatory chemokines (*Cxcl1*,* Ccl2*,* Cxcl3*,* Cxcl2*) in pulmonary MKs of PA-infected mice, compared to the control group (*n* = 3). **H** Representative fluorescence confocal image shows the protein expression of Ccl2 in pulmonary MKs of PA infected mice compared to the control group. **I**, **J** and **K** The KEGG pathway enrichment analysis, GSEA analysis and the heatmap shows the activation of the NF-κb signaling pathway in pulmonary MKs of PA infected mice compared to the control group, up- and down- regulated genes are colored by red and blue for heatmap respectively (*n* = 2). **L** Representative fluorescence confocal image shows the translocation of p65 to the nucleus in pulmonary MKs of PA infected mice compared to the control group. The data shown were mean ± SD. Statistical significance by a two-tailed unpaired t-test is indicated: *P ≤ 0.05; **P ≤ 0.01; ***P ≤ 0.001; ns, not significant (*P* > 0.05)

To further investigate the inflammatory response of pulmonary MKs to PA infection, the lung MK RNA-seq data presented in Fig. 3A were subjected to deeper analysis. The GSEA analysis and the heatmap displayed the upregulated cytokines in PA-infected MKs compared to the sham group (Fig. 4A, B). RT-qPCR analysis further validated the increased expression of four key pro-inflammatory factors (*Tnfα*,* Il1α*,* Il1β*,* Il6*) in pulmonary MKs of infected mice compared to controls (Fig. 4C). Immunofluorescence staining confirmed that TNFα protein expression was elevated in pulmonary MKs of infected mice relative to the control group (Fig. 4D). Furthermore, the GSEA analysis and the heatmap showed significant changes in the chemokine signaling pathway in pulmonary MKs in response to PA infection (Fig. 4E, F). RT-qPCR analysis further validated the increased expression of four key chemokines (*Cxcl1*,* Ccl2*,* Cxcl3*,* Cxcl2*) involved in pro-inflammatory signaling in pulmonary MKs following PA infection (Fig. 4G). Immunofluorescence staining confirmed higher protein expression of Ccl2 in pulmonary MKs after PA infection compared to the control group (Fig. 4H).

Considering that pro-inflammatory and chemotactic signaling pathways in immune cells are likely regulated by the NF-κB signaling pathway, we performed KEGG analysis and found significant activation of the NF-κB pathway in pulmonary MKs following PA infection (Fig. 4I). The GSEA analysis and the heatmap further confirmed the NF-κB signaling activation in PA-infected MKs compared with controls (Fig. 4J, K). Notably, immunofluorescence imaging clearly demonstrated an enhanced translocation of p65 to the nucleus upon PA infection, directly indicating the activation of the NF-κB signaling pathway (Fig. 4L). Overall, these results demonstrated that NF-κB signaling is markedly activated in pulmonary MKs in response to PA infection, leading to increased production of pro-inflammatory factors and chemokines.

### PA surface antigen PcrV specifically triggers MKs activation in the lung

**Fig. 5:**
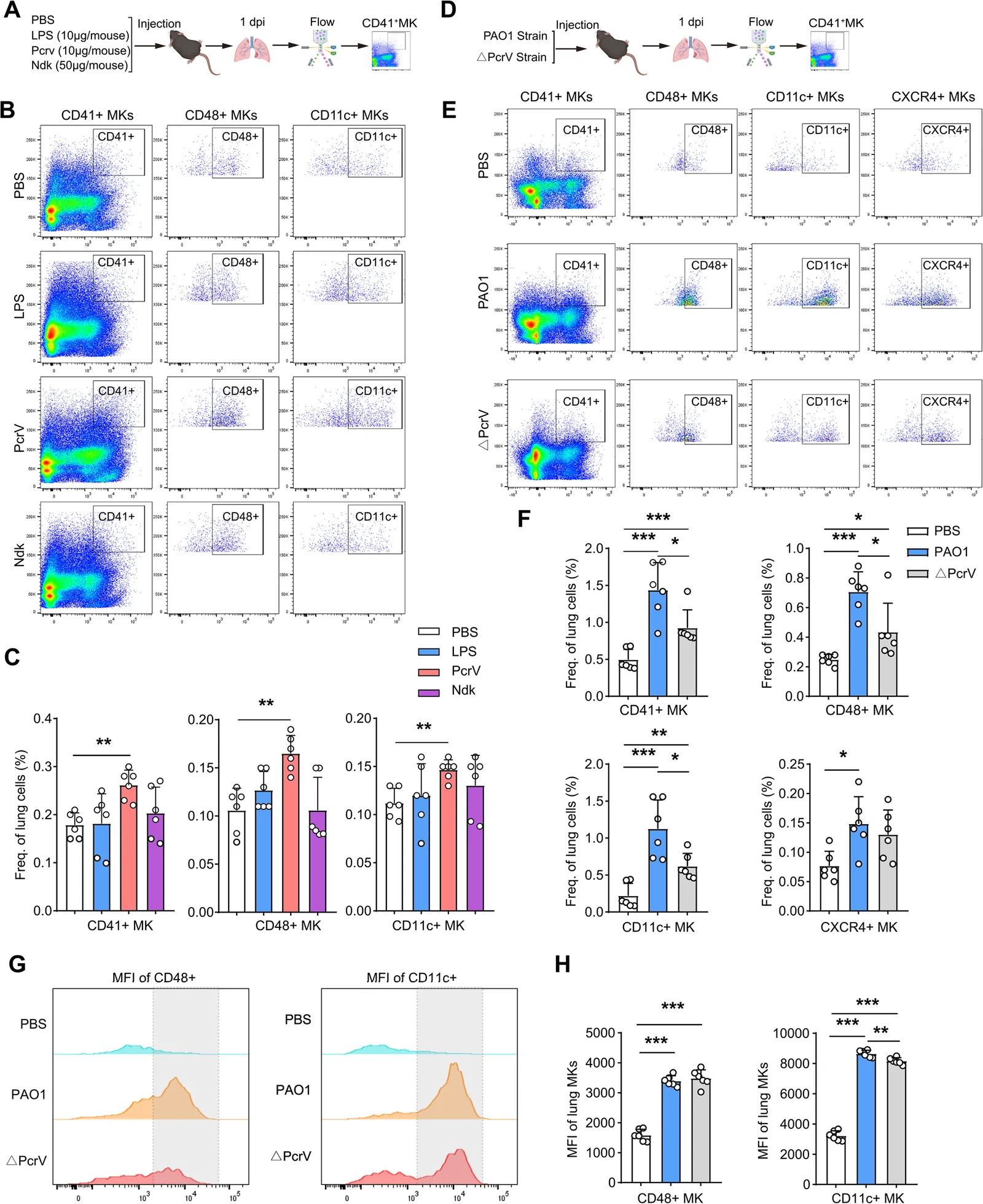
The pulmonary MKs were expended under the stimulation of PA surface antigen PcrV. **A** Schematic illustration of the experimental protocol for PA toxin challenge and flow cytometric characterization of pulmonary MKs. **B** Representative FACS plot of pulmonary MKs and the CD48 + or CD11c + MKs from the LPS, PcrV and Ndk challenged mice compared to the control group. **C** Statistical analysis showing the ratio of pulmonary MKs from LPS, PcrV and Ndk infected mice compared to the control group (*n* = 6). **D** Schematic illustration of the infection procedure via wildtype (PAO1) strain or PcrV mutant (△PcrV) strain, and flow cytometric characterization of pulmonary MKs. **E** Representative FACS plot of pulmonary MKs and the CD48+, CD11c + or CXCR4 + MKs from PAO1 or △PcrV infected mice compared to the control group. **F** Statistical analysis showing the ratio of pulmonary MKs from PAO1 or △PcrV infected mice compared to the control group (*n* = 6). **G** Histograms showing the MFI of CD48 + and CD11c+ pulmonary MKs from PAO1 or △PcrV infected mice compared to the control group. The x-axis denotes MFI. **H** Statistical analysis of the MFI of CD48 + and CD11c+ pulmonary MKs from PAO1 or △PcrV infected mice compared to the control group. The x-axis denotes MFI. The data shown were mean ± SD. Statistical significance by a two-tailed unpaired t-test is indicated: *P ≤ 0.05; **P ≤ 0.01; ***P ≤ 0.001; ns, not significant (*P* > 0.05)

To identify the mediators that stimulate pulmonary MKs in PA-induced pneumonia, mice were separately treated with four PA toxins via intratracheal injection: LPS, pyocyanin, type III secretion protein PcrV, and nucleoside diphosphate kinase (NDK) (Fig. 5A; Supplementary Fig. 3A). After 24 h, lung tissue was analyzed by flow cytometry, and the data showed that challenged with PcrV significantly promoted the expansion of pulmonary MKs. However, LPS, pyocyanin, and NDK did not induce expansion of pulmonary MKs (Fig. 5B and C; Supplementary Fig. 3B, C). Further FACS analysis revealed that LPS treatment led to a significant accumulation of neutrophils, while the number of macrophages and monocytes was lower in the lung tissue compared to the control group (Supplementary Fig. 3F, G). Additionally, treatment with pyocyanin, PcrV, and NDK did not result in significant accumulation of neutrophils, macrophages, or monocytes compared to the control group (Supplementary Fig. 3D-G).

To investigate the specific impact of PcrV on pulmonary MKs, we challenged the mice with a higher dose of recombinant PcrV (25 µg/mouse) (Supplementary Fig. 4A). FACS analysis revealed that the higher dose of PcrV stimulation significantly increased the number of pulmonary MKs, including notable elevations in the proportions of CD48 + and CD11c + MKs, while the CXCR4 + MKs remained inconspicuously (Supplementary Fig. 4B, C). The higher dose of PcrV treatment promoted the entry of pulmonary MKs into an active cell cycle state (Supplementary Fig. 4D). Notably, there were no significant changes in the proportions of other myeloid cells compared to control groups (Supplementary Fig. 4E, F). PcrV is the virulence-associated type III secretion system needle-tip protein of PA, known for its strong immunogenicity. We utilized the wild-type PAO1 strain to construct a PcrV mutant (ΔPcrV) for inducing mouse acute pneumonia (Fig. 5D). Notably, compared with the wild-type PAO1 strain, the ΔPcrV mutant induced a significant decrease in the proportions of total pulmonary MKs, as well as CD48 + and CD11c + MKs (Fig. 5E, F). The histogram and statistical analysis confirmed that the CD48 MFI of MKs in mouse lungs after ΔPcrV and PAO1 infection was similar, while CD11c MFI was slightly lower in the ΔPcrV-infected group but significantly higher than in the control group (Fig. 5G, H). Concurrently, the proportion of proliferative pulmonary MKs in the ΔPcrV group was significantly lower than that in the PAO1 group (Supplementary Fig. 4I, J). Although both infections increased neutrophil infiltration and decreased the macrophage ratio relative to blank controls (Supplementary Fig. 4G, H), the distinct reduction in MK expansion observed upon PcrV deletion explicitly indicates that the PcrV antigen plays a pivotal role in promoting pulmonary MK expansion.

### PcrV-induced NF-κB activation in MKs underpins pro-inflammatory and chemotactic Pathways

**Fig. 6:**
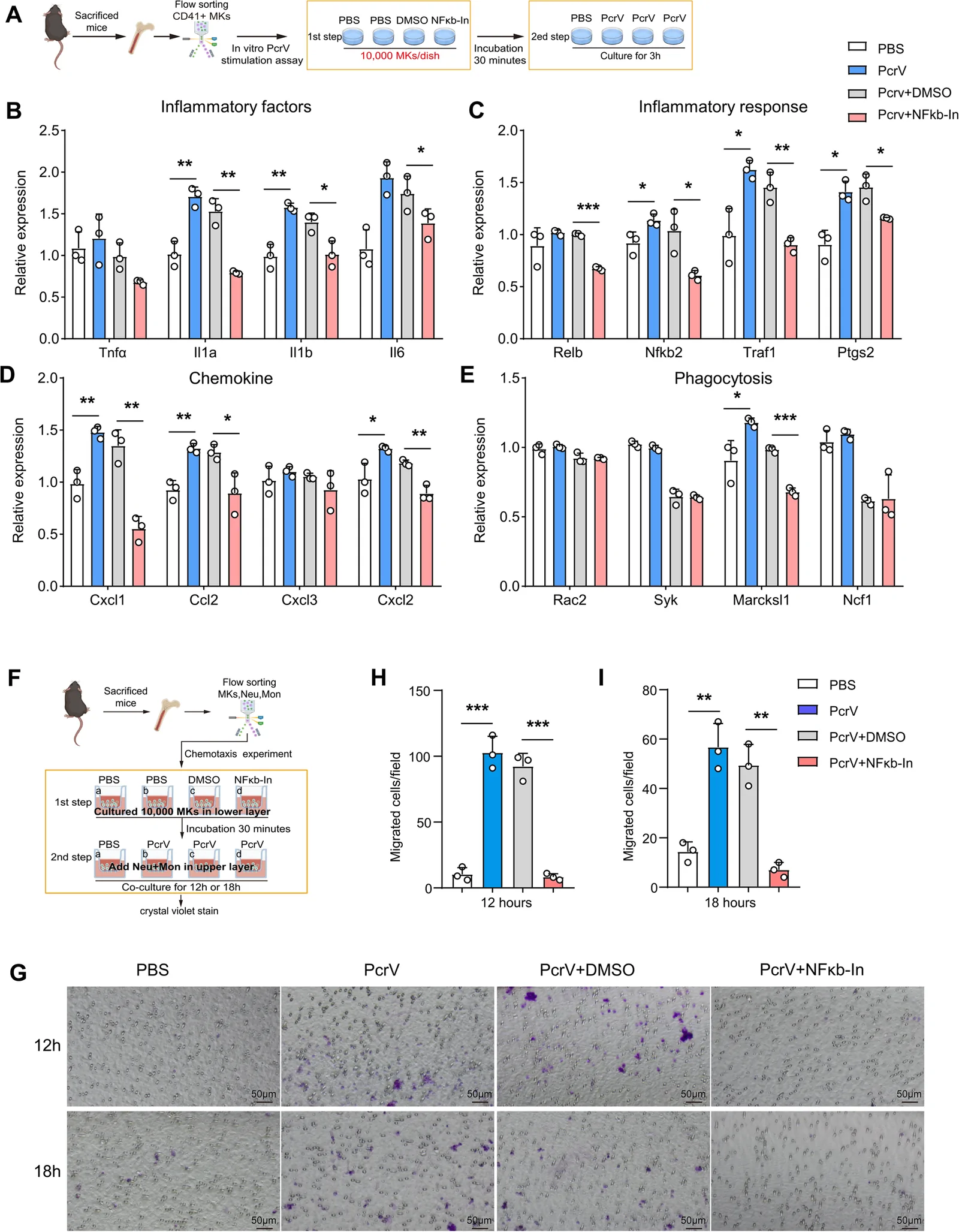
The PA surface antigen PcrV triggered the activation of the NF-κB signaling pathway in MKs. **A** The schematic shows the flow sorting of bone marrow MKs and the procedure of in vitro PcrV stimulation assay. **B** qPCR analysis shows the expression levels of four key pro-inflammatory factors (*TNFα*,* Il1α*,* Il1β*,* Il6*) in flow-sorted MKs following the treatment of PcrV and NF-κb inhibitor in vitro (*n* = 3). **C** qPCR analysis shows the expression levels of four marker genes in inflammatory response signaling (*Relb*,* Nfkb2*,* Traf1*,* Ptgs2*) of flow-sorted MKs following the treatment of PcrV and NF-κb inhibitor in vitro (*n* = 3). **D** qPCR analysis shows the expression levels of four key chemokines (*Cxcl1*,* Ccl2*,* Cxcl3*,* Cxcl2*) in flow-sorted MKs following the treatment of PcrV and NF-κb inhibitor in vitro (*n* = 3). **E** qPCR analysis shows the expression levels of four marker genes in phagocytosis signaling (*Rac2*,* Syk*,* Marcksl1*,* Ncf1*) of flow-sorted MKs following the treatment of PcrV and NF-κb inhibitor in vitro (*n* = 3). **F** The schematic shows the flow sorting of bone marrow MKs, neutrophils and monocytes, and the procedure of chemotaxis experiment in vitro. **G** Chemotaxis assay to verify the ability of PcrV-stimulated MKs to induce neutrophil and monocyte migration. Representative trans-well images showing megakaryocyte-induced neutrophil and monocyte to migrate across the inter-membrane. **H** and **I** Statistical analysis showing the migrated neutrophils and monocytes with crystal violet staining induced by MKs. The data shown were mean ± SD. Statistical significance by a two-tailed unpaired t-test is indicated: *P ≤ 0.05; **P ≤ 0.01; ***P ≤ 0.001; ns, not significant (*P* > 0.05)

To investigate whether PcrV activates MKs through the NF-κB signaling pathway, bone marrow MKs were isolated through FACS sorting and cultured in complete medium. NF-κB inhibitors were then used to block signal transduction, while purified PcrV protein served as the stimulatory factor (Fig. 6A). We found that PcrV stimulation significantly elevated the gene expression of inflammatory factors (TNFα, IL-1α, IL-1β, IL-6) in MKs. However, PcrV cannot induce an increase in these marker genes following NF-κB inhibitor treatment (Fig. 6B). Furthermore, PcrV stimulation activated the inflammatory response pathway in MKs, evidenced by upregulation of RelB, NFKB2, TRAF1, and PTGS2. However, NF-κB signaling inhibition prevented PcrV from effectively activating this pathway (Fig. 6C). Concurrently, PcrV stimulation led to a significant increase in the expression of chemokines (CXCL1, CCL2, CXCL2) in MKs. However, NF-κB signaling inhibition completely abolished this induction (Fig. 6D). Additionally, PcrV stimulation slightly activated the phagocytic pathway in MKs. However, NF-κB inhibition reduced MARCKSL1 activation, and no significant changes were observed in other phagocytosis-related signaling molecules (RAC2, SYK, NCF1) (Fig. 6E). These findings suggested that PcrV stimulation significantly upregulated inflammatory factors, chemokines, and the inflammatory response pathway in MKs, with effects dependent on NF-κB signaling.

NF-κB inhibition abolished these PcrV-induced responses, highlighting the critical role of NF-κB signaling in the activation of inflammation and chemotactic signaling in MKs. To investigate the impact of chemotactic signaling activation in MKs on neutrophil and monocyte migration, bone marrow-derived MKs, neutrophils, and monocytes were sorted and cultured in vitro (Fig. 6F). Chemotaxis assays using trans-well co-culture showed that PcrV-stimulated MKs effectively induced the migration of neutrophils and monocytes toward the MK side (Fig. 6F, G). After inhibiting NF-κB signaling in MKs, no significant chemotactic activity toward neutrophils and monocytes was observed (Fig. 6G). The statistics of the number of migrating cells are shown in Fig. 6H and I. These data indicated that PcrV-stimulated MKs effectively induced neutrophil and monocyte migration in co-culture condition, while NF-κB inhibition prevented this chemotactic activity. Furthermore, in vivo results demonstrated that after PA infection, lung Cxcl1 and Ccl2 levels were significantly lower in MK-depleted mice than in controls (Supplementary Fig. 4K), further suggesting that reduced chemokines account for the decrease in pulmonary myeloid cells in MK-depleted mice.

## Discussion

Recent studies increasingly highlight the pivotal role of MKs in regulating infection-driven inflammation, underscoring their emerging importance far beyond platelet production [10, 29]. As the immune responses to bacterial infections involve the coordinated action of multiple immune cells [34], pulmonary MKs may serve as a modulating member capable of producing inflammatory cytokines and chemokines. A deeper understanding of MKs’ role in pulmonary infections could offer valuable insights into their immune functions and open new therapeutic strategies targeting MK activity in inflammatory diseases. Upon PA infection, pulmonary MKs may function as early responders, leverage the activation of the NF-κB signaling pathway to orchestrate a robust secretome of pro-inflammatory cytokines and chemokines, thereby accelerating the influx of neutrophils and monocytes to the infectious foci. This cellular mobilization is pivotal for mounting an effective host defense, given the indispensable roles of these myeloid lineages in bactericidal clearance. Our findings demonstrate that pulmonary MKs serve as an essential coordinator of the innate immune response, which is required for the prompt clearance of PA within the respiratory tract.

Notably, this accumulation of pulmonary MKs prominently precedes the response in the bone marrow following PA infection. This temporal dissociation suggests that the increased lung MKs may not depend on the *de novo* production and subsequent migration from the bone marrow during the hyperacute phase of infection. Recent groundbreaking studies have redefined the lung as a major site of terminal megakaryopoiesis and a reservoir for hematopoietic progenitors [12]. Based on this, we speculate that the rapid increase in pulmonary MKs may involve two non-exclusive mechanisms: firstly, the rapid in situ maturation of resident megakaryocyte progenitors already present in the lung tissue in response to early inflammatory cues; secondly, the mobilization of a marginal pool of MKs sequestered within the pulmonary microvasculature. Such a localized and rapid response is consistent with the idea that pulmonary MKs may function as early responders or immune sentinels, allowing for the immediate modulation of the immune landscape before the systemic bone marrow-driven compensatory hematopoiesis is fully activated.

The non-canonical immune role of the megakaryocyte-platelet system in pulmonary infections is further supported by evidence regarding circulating platelets. In addition to our findings that pulmonary MKs act as early sentinels via the PcrV-induced NF-κB pathway, previous studies have demonstrated that systemic depletion of circulating platelets impair the lung microvascular integrity [31, 35], also leads to a significant reduction in pulmonary neutrophil recruitment and increased mortality during PA infection [36]. Notably, this previous study and our work provide complementary insights into the host defense landscape. Furthermore, our study utilized Pf4-Cre; iDTR mice combined with intratracheal administration of DT to specifically induce MK depletion within the lung. This localized strategy eliminates pulmonary MKs and platelets at the local condition, offering a more targeted and thorough depletion compared to systemic antibody-mediated platelet depletion. Together, these data underscore that both the “upstream” MKs and their “downstream” platelets are indispensable components of the innate immune response against pathogens in the lung.

The transcriptomic landscape of PA-infected pulmonary MKs revealed a coordinated upregulation of phagocytosis-associated genes, including Syk, Rac2, Ncf1, and Marcksl1. Among these, Marcksl1 exhibited the most prominent increase and was further validated by RT-qPCR. Given the reported role of MARCKSL1 in membrane–cytoskeletal coupling and actin remodeling, its induction may reflect not only an immune-responsive shift, but also infection-induced structural remodeling of pulmonary MKs. Such remodeling may support bacterial uptake, adhesion-related behavior, and inflammatory signaling, and may also intersect with classical MK programs, although this possibility remains speculative and requires further functional validation. In parallel, the upregulation of SYK, RAC2, and NCF1, which are closely linked to phagocytic signaling and cytoskeletal dynamics, is consistent with the enhanced pathogen-interacting phenotype observed in pulmonary MKs after PA infection.

Our study identifies the PcrV as a significant inducer of the immunogenic activation of MKs. It’s well known that specific interactions between pathogens and the immune cells are critical in shaping the outcome of infections [37–39]. PcrV has been well characterized for the formation of the core components of the tip of the type III secretion system, which injects toxins into host cells [40, 41], and its excellent immunogenicity in facilitating the host’s immune response [42]. In this study, we extend this paradigm by demonstrating that PcrV directly triggers MK activation, resulting in the production of pro-inflammatory mediators via the NF-κB signaling pathway.

## Conclusions

This study uncovers a previously underappreciated role of pulmonary MKs in the inflammatory response to PA infection. Traditionally recognized for their role in platelet production, MKs are now shown to be active participants in the lungs’ innate immune defense [10, 29, 43]. While previous studies have largely overlooked the contribution of MKs during bacterial infections, our findings demonstrate that the bacterial antigen PcrV induces an immune-responsive phenotype in MKs via NF-κB–dependent signaling pathways. This phenotype drives the production of pro-inflammatory mediators, thereby shaping the cytokine and chemokine milieu to modulate local lung inflammation. In summary, this study demonstrates that targeted enhancement of pulmonary MKs can trigger an antibacterial innate immune response and clear pathogens, offering a new approach for the treatment of hospital-acquired PA infection.

## Supplementary Information

Supplementary Material 1.

Supplementary Material 2.

## Data Availability

Data supporting the findings of this manuscript could be obtained from the corresponding author upon reasonable request. The RNA-seq data is uploaded to SRA with an accession number: GSE295916.

## Abbreviations

PA: *Pseudomonas aeruginosa (P. aeruginosa)*
MKs: Megakaryocytes
iDTR: Inducible diphtheria toxin receptor
DT: Diphtheria toxin
ROS: Reactive oxygen species
FBS: Fetal bovine serum
IPTG: Isopropyl-β-D-thiogalactopyranoside
PBS: Phosphate-buffered saline
LPS: Lipopolysaccharide
DMSO: Dimethyl sulfoxide
FACS: Fluorescence activated cell sorting
NDK: Nucleoside diphosphate kinase
