# Supplementary material for: Pulmonary megakaryocytes orchestrate host defense via PcrV-induced NF-κB signaling in Pseudomonas aeruginosa infection
Source: Cell Commun Signal. 2026 Jul 20;24:579. doi: 10.1186/s12964-026-03072-y (PMC13712033; doi:10.1186/s12964-026-03072-y)
Supplement: Supplementary file 1 [file 12964_2026_3072_MOESM1_ESM.docx]

**Supplementary Information**

**Contains 4 Supplementary Figures, 1 Supplementary Video and 3 Supplementary Tables**

**Pulmonary Megakaryocytes Orchestrate Host Defense via PcrV-Induced NF-κB Signaling in *Pseudomonas aeruginosa* Infection**

Xiaolong Guo_1,2,†_, Qian Dai_1†_, Xiping Liao_1,3†_, Yannan Ma_1,3_, Hengqian Liu_4_, Junzhi Xiong_1_, Hua Yu_1_, Yuanyuan Li_1_, Kebin Zhang_1_, Jianyun Zhou_1*_, and Hongli Zhou_1,5*_

_1_Clinical Medical Research Centre, the Second Affiliated Hospital, Army Medical University, Chongqing 400037, China.

_2_Laboratory Medicine Department, Zigong Fourth People's Hospital, Sichuan 643000, China.

_3_Department of Gastroenterology, the Second Affiliated Hospital, Army Medical University, Chongqing 400037, China.

_4_Chongqing University Cancer Hospital, School of Medicine, Chongqing University, Chongqing 400030, China.

_5_Lead contact

_†_These authors contributed equally to this work.

Corresponding author: Hongli Zhou, email: [zhouhongli@tmmu.edu.cn](mailto:zhouhongli@tmmu.edu.cn). Jianyun Zhou, email: [zhoujianyun1983@tmmu.edu.cn](mailto:zhoujianyun1983@tmmu.edu.cn)

**Supplementary Figures and video**

**Supplementary Fig. 1 | The mice model of acute pneumonia was successfully induced by PA infection.** (A) Photographs show pulmonary pathological appearance of mice on days 1, 3, 7, 14 and 21 post PA infection compared to the control group. (B) Photographs of CFU counts shows the pulmonary bacteria loads of mice on days 1, 3, 7, 14 and 21 post PA infection compared to the control group, the CFU counts were performed with the tissue lysates following serial dilution. (C-G) FACS analysis the ratio of pulmonary phagocytes, include neutrophils (Neu), monocytes (Mon), macrophages (Mac), eosinophil (Eos) and dendritic cell (DC), of mice on days 1, 3, 7, 14 and 21 post PA infection compared to the control group (n=5). The data shown were mean ±SD. Statistical significance by a two-tailed unpaired t-test is indicated: *P ≤ 0.05; **P ≤ 0.01; ***P ≤ 0.001; ns, not significant (P > 0.05).

**Supplementary Fig. 2** | **Flow Cytometric Gating Strategy and MKs Polyploidy Analysis.** (A) The Gating strategies for pulmonary MKs, including [FSC^hi^ CD41+]: MKs, [FSC^hi^ CD41+CD48+]: CD48+ MKs, [FSC^hi^ CD41+CD11c+]: CD11c MKs, [FSC^hi^ CD41+CXCR4+]: CXCR4 MKs. (B) The Gating strategies for other leukocyte populations, including [CD45+CD11b+]: Myeloid, [CD45+ CD11b+ F4/80- CD86+]: Neutrophils, [CD45+ CD11b+ F4/80+ CD86-] Macrophages, [CD45+CD11b+F4/80-CD86-Ly6c+]: Monocytes. (C) Statistical analysis of ploidy levels in CD41+ MKs from PA-infected mice compared to the control group. (D) The cell counts of FACS for total nuclear cells (TNCs) and other phagocytes in PF4Cre- versus PF4Cre+ mice showed in Figure 2D & 2E. The data shown were mean ±SD. Statistical significance by a two-tailed unpaired t-test is indicated: *P ≤ 0.05; **P ≤ 0.01; ***P ≤ 0.001; ns, not significant (P > 0.05).

**Supplementary Fig. 3 |** **Evaluation of pulmonary innate immune activation induced by various PA toxins.** (A) The schematic shows the Pyocyanine infection procedure and flow analysis for pulmonary MKs. (B) Representative FACS plot of pulmonary MKs and the CD48+ or CD11c+ MKs from Pyocyanine treated mice compared to the control group. (C) Statistical analysis showing the ratio of pulmonary MKs from Pyocyanine treated mice compared to the control group (n=6). (D) Representative FACS plot of pulmonary phagocytes from Pyocyanine treated mice compared to the control group. (E) Statistical analysis showing the ratio of pulmonary phagocytes from Pyocyanine treated mice compared to the control group (n=6). (F) Representative FACS plot of pulmonary phagocytes from LPS, PcrV and Ndk treated mice compared to the control group. (G) Statistical analysis showing the ratio of pulmonary phagocytes from LPS, PcrV and Ndk treated mice compared to the control group (n=6). The data shown were mean ± SD. Statistical significance by a two-tailed unpaired t-test is indicated: *P ≤ 0.05; **P ≤ 0.01; ***P ≤ 0.001; ns, not significant (P > 0.05). (Myeloid: Mye; Neutrophils: Neu; Macrophages: Mac; Monocytes: Mon).

**Supplementary Fig. 4 | The pulmonary MKs were expended under the stimulation of PA surface antigen PcrV.** (A) The schematic shows the PcrV infection procedure and flow analysis for pulmonary MKs. (B) Representative FACS plot of pulmonary MKs and its subset (CD48+, CD11c+ or CXCR4+ MKs) from PcrV treated mice compared to the control group. (C) Statistical analysis showing the ratio of pulmonary MKs from PcrV treated mice compared to the control group (n=8). (D) Representative FACS plot (left) and cumulative % (right) of pulmonary MKs proliferation (CD41+Ki67+) from PcrV treated mice compared to the control group (n=8). (E) Representative FACS plot of pulmonary phagocytes from PcrV treated mice compared to the control group. (F) Statistical analysis showing the ratio of pulmonary phagocytes from PcrV treated mice compared to the control group (n=8). (G) Representative FACS plot of pulmonary phagocytes from PAO1 or △PcrV strains infected mice compared to the control group. (H) Statistical analysis showing the ratio of pulmonary phagocytes from PAO1 or △PcrV strains infected mice compared to the control group (n=6). (I and J) Representative FACS plot (I) and cumulative % (J) of cycling pulmonary MKs (CD41+Ki67+) from PAO1 or △PcrV infected mice compared to the control group (n=6). (K) Cxcl1 and Ccl2 levels in bronchoalveolar lavage fluid from *P.a*-infected mice were measured by ELISA, and the fold changes were calculated. The data shown were mean ± SD. Statistical significance by a two-tailed unpaired t-test is indicated: *P ≤ 0.05; **P ≤ 0.01; ***P ≤ 0.001; ns, not significant (P > 0.05). (Myeloid: Mye; Neutrophils: Neu; Macrophages: Mac; Monocytes: Mon).

**Supplementary Video | The dynamic process of MKs phagocytosing PA.**

**(Video 1A)** Fluorescence confocal in vitro live-cell imaging was used to capture the dynamic process of pulmonary MKs phagocytosing PA. PA endogenously expressing GFP was labeled with green fluorescence, while FACS-sorted pulmonary MKs were labeled with red fluorescence via SiR-Actin dye.

| Strains | Description of Strains |
| --- | --- |
| PA PAO1 | Wild-type laboratory strain. |
| PA PAO1 (λ-Red) | PAO1 transformed with a λ-Red recombination system; Cb^R^. |
| PAO1 ΔPcrV | Deletion of PcrV in PAO1; Tet^R^, Cb^R^. |
| JM109 | E. coli gene expression strain. |
| BL21 (DE3) | E. coli gene expression strain. |
| DH5α | E. coli cloning strain. |

**Supplementary table 1. The Strains Used in Study.**

Note: Cb^R^: carbenicillin resistance. Tet^R^: Tetracycline resistance.

| **Supplementary table 2.** **The Antibodies Used in Study.** | | | |
| --- | --- | --- | --- |
| Antibodies | Conjugated fluorescent | Source | Cat. Number |
| CD41 | APC | Biolegend, USA | Cat#133914 |
| CD11c | Brilliant Violet 421 | Biolegend, USA | Cat#117343 |
| CXCR4 | PE | Biolegend, USA | Cat#146506 |
| CD48 | APC/Cyanine7 | Biolegend, USA | Cat#103432 |
| CD86 | FITC | Invitrogen, USA | MHCD8601 |
| CD45 | APC/Cyanine7 | Biolegend, USA | Cat#103115 |
| CD11b | PerCP/Cyanine5.5 | Biolegend, USA | Cat#101228 |
| Ly6G | FITC | Biolegend, USA | Cat#127645 |
| F4/80 | APC | Biolegend, USA | Cat#123116 |
| LY6C | Brilliant Violet 421 | Biolegend, USA | Cat#128032 |
| Zombie | PE | Invitrogen, USA | L34987 |
| Ki67 | FITC | Invitrogen, USA | Cat#11-5698-82 |

| **Supplementary table 3.** **Primer Sequences Used in Study. (F: forward, R: reverse)** | | | |
| --- | --- | --- | --- |
| Species | Gene |  | Sequences（5'to3'） |
| Mus musculus | Traf1 | F | GTGAGTGAGATGGGCATGTTT |
| Mus musculus | Traf1 | R | GAGTGGCAAGAAGGCTGGAT |
| Mus musculus | Nfkb2 | F | GGCCGGAAGACCTATCCTACT |
| Mus musculus | Nfkb2 | R | CTACAGACACAGCGCACACT |
| Mus musculus | Ptgs2 | F | TGAGCAACTATTCCAAACCAGC |
| Mus musculus | Ptgs2 | R | GCACGTAGTCTTCGATCACTATC |
| Mus musculus | Relb | F | CCGTACCTGGTCATCACAGAG |
| Mus musculus | Relb | R | CAGTCTCGAAGCTCGATGGC |
| Mus musculus | Il1b | F | GCAACTGTTCCTGAACTCAACT |
| Mus musculus | Il1b | R | ATCTTTTGGGGTCCGTCAACT |
| Mus musculus | Gapdh | F | GGTGAAGGTCGGTGTGAACG |
| Mus musculus | Gapdh | R | CTCGCTCCTGGAAGATGGTG |
| Mus musculus | Il1a | F | CGAAGACTACAGTTCTGCCATT |
| Mus musculus | Il1a | R | GACGTTTCAGAGGTTCTCAGAG |
| Mus musculus | Il6 | F | TAGTCCTTCCTACCCCAATTTCC |
| Mus musculus | Il6 | R | TTGGTCCTTAGCCACTCCTTC |
| Mus musculus | Cxcl1 | F | CTGGGATTCACCTCAAGAACATC |
| Mus musculus | Cxcl1 | R | CAGGGTCAAGGCAAGCCTC |
| Mus musculus | Ccl2 | F | TTAAAAACCTGGATCGGAACCAA |
| Mus musculus | Ccl2 | R | GCATTAGCTTCAGATTTACGGGT |
| Mus musculus | Cxcl2 | F | CCAACCACCAGGCTACAGG |
| Mus musculus | Cxcl2 | R | GCGTCACACTCAAGCTCTG |
| Mus musculus | Rac2 | F | GACAGTAAGCCGGTGAACCTG |
| Mus musculus | Rac2 | R | CTGACTAGCGAGAAGCAGATG |
| Mus musculus | Syk | F | CTACCTGCTACGCCAGAGC |
| Mus musculus | Syk | R | GCCATTAAGTTCCCTCTCGATG |
| Mus musculus | Marcksl1 | F | CAATGGAGACTTAACCCCCAAG |
| Mus musculus | Marcksl1 | R | GGCCACTCAATTTGAAAGGCT |
| Mus musculus | Ncf1 | F | ACACCTTCATTCGCCATATTGC |
| Mus musculus | Ncf1 | R | TCGGTGAATTTTCTGTAGACCAC |
